# Enlighten the non-illuminated region by phase segregation of mixed halide perovskites

**DOI:** 10.1038/s41377-022-01019-9

**Published:** 2022-10-26

**Authors:** Yan Lv, Junran Zhang, Xiaolong Chen, Lin Wang

**Affiliations:** 1 School of Flexible Electronics (Future Technologies) & Institute of Advanced Materials, Key Laboratory of Flexible Electronics, Jiangsu National Synergetic Innovation Center for Advanced Materials, School of Physical and Mathematical Sciences, Nanjing Tech University, Nanjing, 211816 China; 2 Department of Electrical and Electronic Engineering, Southern University of Science and Technology, Shenzhen, 518055 China

**Keywords:** Optics and photonics, Optical physics

## Abstract

The well-known ion migration in mixed halide perovskites has been intensely investigated within the area under uniform light illumination. Here, the authors demonstrate that the anion segregation in these materials is a nonlocal effect of which the ion redistribution may occur at a macroscopic or mesoscopic scale beyond.

## Abstract (web summary)

Ions in mixed halide perovskite migrate to the non-illuminated region to form a macroscopic ring structure under laser irradiation, which is reversible after removing the light.

## Graphical abstract

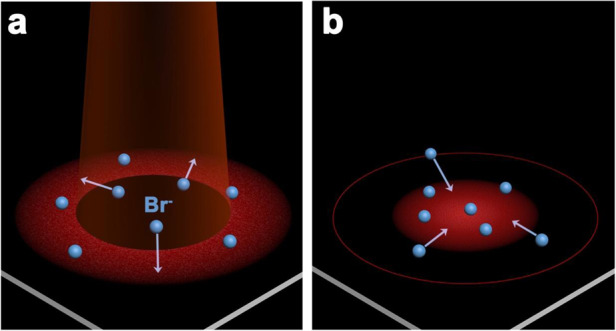

Ion migration is an inherent property of halide perovskites, stemming from their soft lattice structure and ionic conductivity. Upon illumination, the migrating anions in mixed halide perovskites lead to the formation of iodine- and bromine-rich domains, which is known as photo-induced phase segregation or Hoke Effect^1^. Carriers excited by light tend to funnel and recombine in the iodine-rich regions with a smaller energy bandgap^2^, causing an observable redshift in the photoluminescence (PL) spectra. Once the illumination is removed, the system will gradually revert back to its initial mixed phase, driven by the concentration gradient^3^ or the mixing entropy^4^.

Despite this commonly observed phenomenon, the formation mechanism of these separated phases still remains unclear. Several models have been proposed to solve the puzzle. For example, the thermodynamic driving force, provided either by the incident light or by the bandgap reduction of iodide-rich phase^5^, could induce halide migration once the kinetic barriers are overcome. In the polaron picture, the excited charge carries interacts with the soft lattice by strong electron-phonon coupling. It is proposed that the resulting strain induced by polaron formation is sufficient to drive the anion separation and the nucleation of iodine-rich domains^6^. Halide vacancies may also play a critical role in the phase segregation process due to their low formation energies. Under laser irradiation, vacancy hopping can be activated to drive the ion motion in mixed halide perovskites^4^. Another trap-related model suggests that the local electric field generated by surface-trapped charge carriers can serve as the initial driving force for phase segregation^7^. The unbalanced space charge distribution along grain boundaries can produce a similar effect in polycrystalline materials^8^.

A recent publication in *Light: Science & Applications* by Sun et al. provided a brand-new insight into the phase segregation process in MA_0.17_FA_0.83_Pb(I_0.5_Br_0.5_)_3_ polycrystalline thin films^9^. They revealed that the photo-induced ion migration may occur at a macroscopic scale up to 2 mm, indicating their potentials for practical applications. By performing spatially resolved confocal PL mapping at the wavelength of 670 and 790 nm simultaneously for both the illumination and recovery processes, the authors were able to establish a comprehensive picture of the photo-induced variations in the material. It should be noted that the PL scanning area was ~10 times larger than the laser beam size, far beyond the illuminated region. Optical transmission measurements and time-resolved PL mapping were further involved to gain a deeper understanding.

In addition to the expected PL redshift from ~670 to ~790 nm within the laser spot, a ring-like structure with the initial alloy state emission was concurrently observed surrounding the illuminated area (Fig. 1). The intensity was significantly enhanced in the ring exterior, resulting in a donut-like distribution in the PL mapping centered at 670 nm. The steady ring structure arises from two counter-like effects: the light-induced outward diffusion of Br ions and the Coulombic interaction between the Br ion deficit center and surplus ring. The former effect should originate from vacancy-assisted ion migration, as evidenced by the remarkably stronger PL emission along with slower PL decay. Furthermore, the vacancy origin was validated by the density of free Br ions estimated from the measured potential of ~0.4 V between the center and ring, in consistent with the previously reported values. In the case of annular laser excitation, a bright outer ring and a bright inner disk appeared, unambiguously showing the unlimited direction of the light-induced Br ion motion. It is inspiring that the migration could be truncated by artificial scribing, suggesting a possibility to modulate the ion motion in the designed way. In accordance with the defect-related formation of the ring, the illuminated area with red-shifted emission originated from the newly-formed defects, deduced by the evolution time scale, transmission signals and PL lifetime. When the light was removed, the displaced Br ions would diffuse back to the center but take energetically more favorable sites to form better crystallinity. The reverse process was much slower compared to the formation since it mainly relies on the restoring force provided by the remaining electric field. Last but not least, a damped ultra-low-frequency oscillation of ion motion in solid was monitored for the first time during over one hundred hours of recovery.

**Fig. 1:**
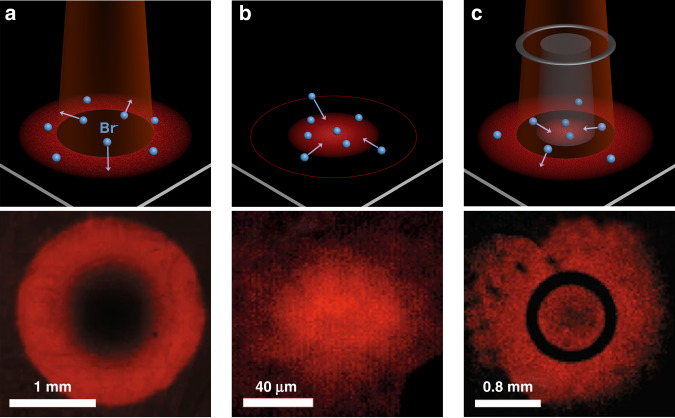
Schematic illustration and experimental photoluminescence mapping of Br ion migration. **a** Under illumination with a beam size of ~1 mm, **b** when the illumination is removed, and **c** under illumination with an annular laser beam. All the experimental results were detected at the wavelength of 670 nm

Inspired by the pioneering achievements in this work, more strategies to modulate ion migration and novel applications based on mixed halide perovskites can be envisioned. Efforts have been done in previous work to inhibit this phenomenon by compositional and structural optimization as well as defect reduction^10^. Future attempts to control this effect can be done via an external electric field or artificial scribing, as implicated by this work. On the other hand, it will provide new opportunities for expanding the functional applications of mixed halide perovskites, if we can take advantage of the generality of light-induced halide segregation, which is rarely applied in practice^11^. For example, a built-in voltage between the ring and the center, resulting from the ion displacement in this work, can be applied in energy storage, as long as the voltage magnitude and the long-term stability can be enhanced. Furthermore, via precise control of the nonlocal halide distribution by ion migration, new opportunities for the applications of perovskites in optical sensing, switching, memory, and security can be expected.

## References

[1] Hoke ET (2015). Reversible photo-induced trap formation in mixed-halide hybrid perovskites for photovoltaics. Chem. Sci..

[2] Yoon SJ (2016). Tracking iodide and bromide ion segregation in mixed halide lead perovskites during photoirradiation. ACS Energy Lett..

[3] deQuilettes DW (2016). Photo-induced halide redistribution in organic-inorganic perovskite films. Nat. Commun..

[4] Barker AJ (2017). Defect-assisted photoinduced halide segregation in mixed-halide perovskite thin films. ACS Energy Lett..

[5] Draguta S (2017). Rationalizing the light-induced phase separation of mixed halide organic-inorganic perovskites. Nat. Commun..

[6] Bischak CG (2017). Origin of reversible photoinduced phase separation in hybrid perovskites. Nano Lett..

[7] Zhang HC (2019). Phase segregation due to ion migration in all-inorganic mixed-halide perovskite nanocrystals. Nat. Commun..

[8] Tang XF (2018). Local observation of phase segregation in mixed-halide perovskite. Nano Lett..

[9] Sun XX, Zhang Y, Ge WK (2022). Photo-induced macro/mesoscopic scale ion displacement in mixed-halide perovskites: ring structures and ionic plasma oscillations. Light Sci. Appl..

[10] Slotcavage DJ (2016). Light-induced phase segregation in halide-perovskite absorbers. ACS Energy Lett..

[11] Mao WX (2021). Light-induced reversal of ion segregation in mixed-halide perovskites. Nat. Mater..

